# Inverted tunnel scleral fixation technique for dropped intraocular lens complex: the double-flanged suture method

**DOI:** 10.5935/0004-2749.2024-0190

**Published:** 2025-04-07

**Authors:** Selim Doganay, Duygu Erdem, Derya Doganay, Mehmet Omer Kiristioglu

**Affiliations:** 1 Department of Ophthalmology, Bursa Uludag University School of Medicine, Bursa, Turkey; 2 Department of Ophthalmology, Elazıg Fethi Sekin City Hospital, Elazig, Turkey; 3 Department of Ophthalmology, Bursa Yuksek Ihtisas Training and Research Hospital, Bursa, Turkey

**Keywords:** Scleral fixation, Intraocular lens dislocation, Ophthalmologic surgical procedures, Sutures, Intraocular lens, Lens subluxation

## Abstract

**Purpose:**

The aim of this study is to describe a minimally invasive and atraumatic technique for managing the polypropylene suture-assisted scleral fixation of intraocular lens-capsular bag complex or artificial iris-intraocular lens complex for repositioning late luxated or subluxated intraocular lens-capsular bags and artificial iris-intraocular lens complexes.

**Methods:**

In this retrospective and observational study, we evaluated 11 patients, including 10 patients with capsular bag-intraocular lens complex subluxation or luxation into the vitreous cavity and 1 patient with an aniridia-intraocular lens complex. A single senior surgeon performed the procedures. After anesthesia, a 4 × 4 mm conjunctival peritomy was created, and a 6-0 polypropylene suture was passed through the sclera marked 2.0 mm posterior to the limbus. The suture ends were cauterized into a flange under 0.5 mm and inserted inversely into a scleral tunnel, concealed within a 2-mm scleral tunnel to ensure secure intraocular lens positioning.

**Results:**

We analyzed 11 patients with dislocated or dropped capsular bag-intraocular lens complexes. The patients’ median age was 67 (range 44-78) years, with a median follow-up of 10 (range: 4-16) months. There were 8 (72%) men and 3 (27%) women. Conjunctival peritomy was performed in 4 (36%) patients. Predominantly, preoperative diagnoses indicated 7 (63%) patients with dislocated capsular bag-intraocular lens complexes. The capsular bag-intraocular lens complexes were centralized in all patients, and optical coherence tomography confirmed accurate suture positioning within the sclera. No suture-related complications were observed throughout the follow-up period, and no vision-threatening complications were reported during the postoperative follow-up.

**Conclusions:**

Our technique provides a simple, effective solution for treating decentralized or dislocated capsular bag-intraocular lens complexes, eliminating the need for complex interventions such as large corneal wounds, scleral flaps, intraocular lens exchange, and intraocular lens externalization.

## INTRODUCTION

Dislocation of the capsular bag-intraocular lens (CB-IOL) complex is recognized as a delayed complication after cataract surgery^(1)^. In cases where lens dislocations result in visual impairments, recentralizing the intraocular lens (IOL) is recommended. Current surgical approaches often favor preserving and repositioning the original lens rather than replacing it^(2)^. Various techniques, both with and without sutures, have been documented for this purpose^(3^-^6)^. The concept of sutureless IOL fixation was initially introduced by Schariot and Pavlidis in 2007^(7)^. Today, the most prevalent intrascleral lens fixation methods include the glued IOL and Yamane techniques, which eliminate the need for sutures and reduce the risk of postoperative complications related to sutures^(8)^.

Nonetheless, sutured techniques have experienced a resurgence, especially in repositioning subluxated or luxated IOLs. For instance, Canabrava et al. introduced the double-flanged technique, in which 5/0 polypropylene sutures are used to stabilize dislocated IOLs and capsular tension ring or segments^(9^,^10)^. Furthermore, Ehud Assia et al. used a 6/0 polypropylene suture for repositioning subluxated IOLs, which is referred to as adjustable flanged scleral fixation^(11)^. Canabrava et al. recommended cauterizing a knob and placing the flange in a scleral tunnel to prevent complications and conceal it under the conjunctiva and Tenon’s capsule, whereas Assia et al. suggested a sub-Tenon approach^(9^,^11)^.

After scleral fixation, patients may experience complications such as suture breakage, erosion, and exposure. The chosen suture material and the specific surgical technique used play a significant role in the likelihood of these issues^(3)^. In general, 5/0 and 6/0 polypropylene sutures are considered safe for intraocular use^(12)^.

In this study, we modified the scleral loop fixation or closed-loop haptic technique using a 6/0 polypropylene suture. Our technique involves accessing the sclera through the same entry point to create a loop, followed by using the inverted tunnel technique to embed the flange within the sclera. Our aim was to reduce the incidence of suture-related complications.

## METHODS

We conducted a retrospective, single-center, observational study of patients who underwent the above mentioned surgical technique between January 1, 2019, and July 13, 2022. Patients were identified using a query in the hospital’s electronic information system.

Patients were recruited after obtaining approval from the regional ethics committees. Informed consent was obtained from all patients. The tenets of the Declaration of Helsinki were followed.

Dislocation of the CB-IOL complex was observed in seven patients. Specifically, in three of these patients, the CB-IOL complex luxated into the vitreous cavity. Among these patients, two patients had previously undergone pars plana vitrectomy (PPV) due to retinal detachment or diabetic retinopathy, and one patient had undergone pseudoexfoliation without any previous PPV.

For patients with subluxated CB-IOL complexes, the surgical approach was determined based on the position of the IOL haptic. In two patients, surgery involved conjunctival peritomy, as outlined in our surgical method; however, in five patients, the procedure was performed without opening the conjunctiva. In two vitrectomized patients with luxated complexes in the vitreous cavity, we performed a 25-gauge (g) PPV. The procedure involved chandelier illumination and filling the vitreous cavity with perfluorocarbon liquid up to the iris level, followed by the application of our surgical technique to the floating CB-IOL complex at the pupil level. In these instances, the surgical approach involved conjunctival peritomy in one patient, whereas in the other two patients, the procedure commenced with opening the conjunctiva.

In a particular patient with aphakia and total aniridia, late subluxation occurred after the accidental removal of one of the three flange sutures at an external center. These sutures were initially fixed to the sclera using the Canabrava flange technique as described previously^(9^,^10)^. Our technique was used to address this subluxation. This patient also presented with total retinal detachment in addition to artificial iris-IOL complex subluxation. Initially, the lens sutures, which were placed at three points using the Canabrava technique, were removed in sequence. Then, using our fixation method, the artificial iris-IOL complex was secured to the sclera at three points, involving the opening of the conjunctiva, after which PPV was successfully performed.

### Surgical technique

**Figure 1:**
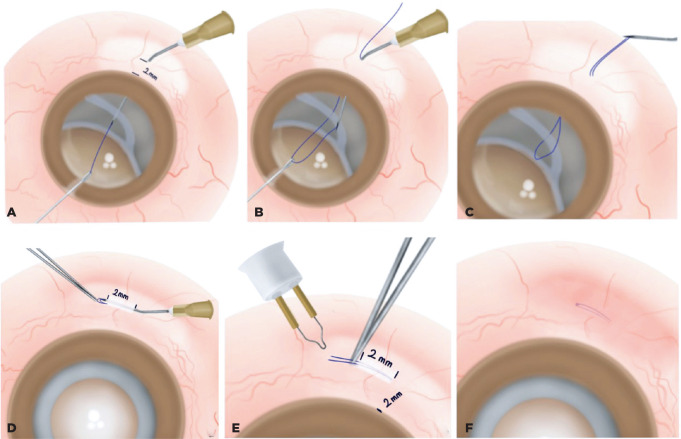
Schematic of important steps of the surgical procedure. A. A 27-gauge (g) needle was used to access the anterior chamber 2 mm from the limbus, after which the end of a 6-0 polypropylene suture was passed through the needle’s bevel IOL haptic. B. Using forceps through a corneal side port, one end of the polypropylene suture was pulled over the IOL, and the 27-g needle was subsequently removed via loop formation. Pulling the suture ends helps centralize the IOL, whereas threading the suture through the needle tip helps create a 2-mm scleral tunnel to conceal the suture. C. Both suture ends are threaded through the tip of the 27-g needle. D. The suture ends penetrated through the sclera, creating an inverted scleral tunnel via a 27-g needle. E. The suture ends were pulled to the outside of the tunnel to create a flange with cautery. F. The flanged suture ends are embedded into the sclera.

All procedures were performed by a single senior surgeon. Topical 1% tropicamide was applied twice, 30 min before administering retrobulbar anesthesia. Standard aseptic protocols were followed, including the use of a 10% povidone-iodine solution periocularly and a 5% povidone-iodine instillation. After administering standard ophthalmic local anesthesia and preparing the surgical site, a conjunctival peritomy of approximately 4 × 4 mm was created in two quadrants aligned with the IOL haptic position (the surgical technique can also be performed without opening the conjunctiva). Next, the sclera was marked 2.0 mm posterior to the limbus (adjustable based on the IOL haptic position). A 6-0 polypropylene suture was inserted into the tip of a 27-gauge needle without entering the eye. Then, a 27-gauge needle threaded with a 6-0 polypropylene suture was passed through the marked sclera, posteriorly to anteriorly under the haptic of the CB-IOL complex. One end of the polypropylene suture was grasped and pulled over the IOL using microforceps through the corneal side port. The suture was then pulled out through the corneal side port, and the 27-gauge needle was completely removed (Figure 1A), reinserted at the same entry point, and directed above the haptic at that time (Figure 1B). Microforceps were used to guide one end of the suture into the needle’s syringe lumen, after which the needle was retracted. This approach secured the haptic from both sides, providing a pivot point for easy manipulation of the haptic (Figure 1C). Pulling the suture ends centralized the IOL. To prevent conjunctival erosion and ensure long-term stabilization, the suture ends were threaded through the tip of the 27-g needle, creating a 2-mm inverted scleral tunnel to conceal the suture (Figure 1D). The suture ends were cauterized using an ophthalmic cautery device (Accu-Temp Cautery, Beaver Visitec) to form a flange <0.5 mm in diameter (Figure 1E). The flanged ends were then pressed back and secured within the scleral tunnel via a 27-g needle tip (Figure 1F). The conjunctival peritomy was closed using 8/0 polyglactin sutures (Vicryl). This procedure was repeated using the same technique and a 180° opposite needle to achieve precise centration and axial stability of the IOL. At the end of the surgery, intracameral triamcinolone acetonide was administered to check for vitreous prolapse, and anterior vitrectomy was performed when the vitreous strands were stained. A case with a dropped CB-IOL complex and subsequent scleral fixation technique (Uludag technique) is demonstrated in Video 1.

## RESULTS

The average age of the patients was 67 (range 44-78) years, and the mean follow-up period was 10 (range: 4-16) months. There were 8 (72%) male and 3 (27%) female patients. There were no vision-threatening complications throughout the postoperative follow-up during the perioperative period. Demographic data of the patients, along with their preoperative surgical diagnoses and the types of IOLs used, are shown in Table 1. Conjunctival peritomy was performed in 4 (36%) patients, whereas in 7 (63%) patients, the conjunctiva was not opened. Preoperatively, 7 patients (63%) were diagnosed with a dislocated IOL capsular complex, and 3 patients (27%) had a dropped IOL capsular complex. Among these cases, three-piece and single-piece lenses were detected in 7 (63%)and 3 (27%) patients, respectively. Furthermore, 1 patient (9%) had a dislocated artificial iris-IOL complex.

**Table 1:** Demographic features of the 11 patients

Case	Age	Sex	Follow-up period (month)	Preoperative diagnosis	IOL type	IOL brand	Prior PPV	Conjunctival peritomy
1	58	Male	14	Dislocated IOL-capsule	Three-piece	Alcon	-	Applied
2	44	Male	16	Dislocated artificial iris-IOL	Artificial iris	Reper	+	Applied
3	73	Female	9	Dropped IOL-capsule	Three-piece	J&J	+	N/A
4	56	Male	11	Dislocated IOL-capsule	Three-piece	Alcon	-	N/A
5	72	Male	6	Dislocated IOL-capsule	One-piece	Alcon	-	N/A
6	65	Male	13	Dislocated IOL-capsule	One-piece	Alcon	-	N/A
7	68	Male	15	Dropped IOL-capsule	Three-piece	J&J	+	Applied
8	63	Female	4	Dropped IOL-capsule	One-piece	Alcon	+	N/A
9	75	Female	7	Dislocated IOL-capsule	Three-piece	Alcon	-	Applied
10	78	Male	8	Dislocated IOL-capsule	Three-piece	J&J	-	N/A
11	67	Male	10	Dislocated IOL-capsule	Three-piece	J&J	-	N/A

The IOLs used in the procedures included three-piece hydrophobic acrylic IOLs, viz., AcrySof MA60AC (Alcon, Fort Worth, Texas, USA) and Sensar AR40 (Johnson & Johnson Vision, Irvine, California, USA). A single-piece hydrophobic acrylic IOL, AcrySof SA60AT (Alcon, Fort Worth, Texas, USA), was also used. The artificial iris-IOL complex used was Reper^®^ Model C (Nizhny Novgorod, Russia).

Remarkably, no patients exhibited tilt, and optical coherence tomography confirmed correct suture positioning within the sclera across all patients. No suture-related complications were detected during the follow-up.

## DISCUSSION

Decentralization of IOLs is a serious postoperative complication of cataract surgery that generally occurs in the late stage and is termed late-in-the-bag dislocation. This process involves spontaneous dislocation for more than 3 months due to the gradual decrease in the stability of the zonules^(13)^. These IOLs may become decentralized outside the pupil or descend into the vitreous cavity, particularly in patients with zonular diseases such as pseudoexfoliation^(14^,^15)^.

Addressing IOL dislocation is a significant challenge despite the numerous techniques that have been described, and optimal management is still under debate^(4^,^16)^. The choice of surgical technique generally depends on the nature of the dislocation, the type of IOL, and the availability of surgical instruments. Although multiple methods have been established, sutured techniques are becoming increasingly preferred in modern ophthalmic practices^(17)^.

Our technique can be effectively described in two distinct phases. Initially, we adapted and refined Assia’s adjustable polypropylene method^(11)^. We hypothesize that entering the sclera from the same point improves the adjustment of the IOL’s stability and the tension of the polypropylene suture. Next, we efficiently embedded the suture into the sclera with inverted tunnel only. Hence, it becomes possible to complete the sclerotomy step without the need for creating a scleral flap or using additional sutures, unlike the scleral fixation methods described in the literature^(18^,^19)^.

**Figure 2:**
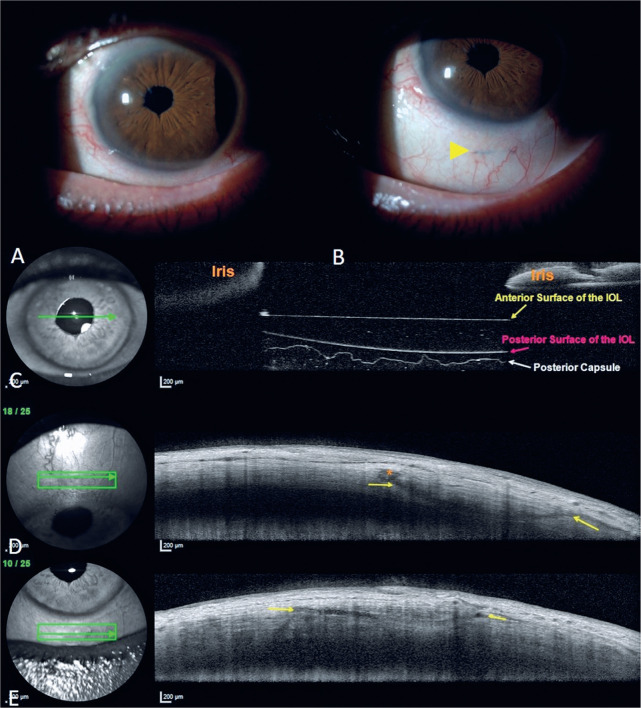
Anterior segment photograph and anterior segment optical coherence tomography (AS-OCT) images. A. The anterior segment photograph reveals no signs of pupil distortion or decentralization. B. A polypropylene suture embedded in the sclera is indicated by the yellow arrow. Due to its deep burial within the sclera, it appears notably pale. C. The AS-OCT image, captured through the pupillary aperture, delineates the front surface, back surface, and posterior capsule of the intraocular lens (IOL). The absence of tilt and its nearly perfect perpendicular alignment to the optical axis are clearly visible. D. In the superior scleral quadrant, a buried suture is marked between two yellow arrows. The conjunctiva is seen to be fully closed over the suture without direct contact or exposure. An orange asterisk highlights the thick flange at the end of the suture. E. In the inferior scleral quadrant, another embedded suture is visualized between the yellow arrows.

The sutures or flanges of scleral fixated IOLs may erode the conjunctiva^(20^,^21)^. Moreover, the flange may expose and cause ocular surface pathogens to translocate to the vitreous cavity, potentially causing sight--threatening endophthalmitis^(22)^. The uniqueness of our approach lies in the embedding with the inverted scleral tunnel, where our goal was to reduce the incidence of postoperative complications such as suture erosion and flange exposure, thereby minimizing suture-related issues and reduce scleral manipulation^(23)^. Throughout the follow-up, our patients showed no suture-related complications. Postoperative anterior segment optical coherence tomography of the sclerotomy area revealed no signs of conjunctival erosion or haptic dislocation (Figure 2). Similar to Assia’s method, our technique allows easy adjustment of the IOL position with sutures threaded through the inverted scleral tunnel.

Our technique uses an open conjunctival approach in cases where subconjunctival chemosis and hemorrhage develop during peribulbar anesthesia. For seven patients with a normal conjunctiva that would not interfere with the technique, surgeries were performed without opening the conjunctiva. However, in cases of luxated or subluxated IOLs requiring scleral fixation, we found it essential to expose the sclera by opening the conjunctiva whenever the sclera was not fully visible. This ensures the safety and reliability of the fixation procedure.

One of our cases involved scleral fixation of the Reper-IOL complex. The literature describes case reports regarding the fixation of the Reper-IOL complex using the Canabrava method and scleral fixation using Gore-Tex sutures^(24^,^25)^. As highlighted by Ozcan and Aydamirov, although the Canabrava method is effective, the primary issue lies in the direct insertion of sutures into the sclera without burying the flanges^(24)^. Our method addresses this problem by both encompassing the flanges and burying them into the sclera with an inverted tunnel.

The primary advantages of our technique include its minimally invasive nature, lack of complex instruments, and relatively short surgical duration. Moreover, the most crucial aspect of the procedure is accurately penetrating the sclera to an appropriate depth and effectively securing the flange within the tunnel postcauterization. Previous research has demonstrated that our method of cauterizing a 1-mm-long suture tip is highly effective^(26)^. Meticulous care is required during the stages of suture tip cauterization, and the 27-g needle penetrates the sclera to the correct depth, with a significant amount of time devoted to these steps. Adjusting the suture tension during the application of our technique is critical, which otherwise might result in centralization problems after surgery. Another potential limitation is the unknown long-term effect. Studies have reported that the incidence of suture breakage with polypropylene sutures within the first 10 years postsurgery is approximately 26.2%^(27^,^28)^.

To summarize, our technique represents a straightforward and effective surgical procedure that can be performed without complex surgical procedures. Furthermore, threading the suture through the scleral tunnel and fully embedding the flange within it serve as safeguards against both haptic and suture-related complications. We acknowledge the contributions of Yamane and Canabrava for their pioneering work in the flange technique in this field^(8^,^9)^. Nonetheless, a broader series of case studies and a longer follow-up period are essential to validate the stability of the fixation and evaluate any associated complications.
